# A new troglobitic species of *Tychobythinus* from Sicily with notes on some Italian species of the genus (Coleoptera, Staphylinidae, Pselaphinae)

**DOI:** 10.1371/journal.pone.0316855

**Published:** 2025-02-19

**Authors:** Giorgio Sabella, Marco Mariano Interlandi, Giuseppe Nicolosi

**Affiliations:** 1 Department of Biological, Geological and Environmental Sciences, Section Animal Biology, University of Catania, Catania, Italy; 2 Legambiente Sicilia, Nature Reserve “Grotta di Sant’Angelo Muxaro” Management Authority, Sant’Angelo Muxaro (AG), Italy; 3 Molecular Ecology Group (MEG), Water Research Institute (IRSA), National Research Council (CNR), Verbania, Pallanza, Italy; Chulalongkorn University, THAILAND

## Abstract

A new troglobitic species of the subfamily Pselaphinae (Coleoptera: Staphylinidae), *Tychobythinus muxari* **n**. **sp**., is described from Sicily (Ciavuli cave, Sant’Angelo Muxaro, Agrigento). Major diagnostic features are illustrated based on both male and female specimens. The new species shows some adaptations to cave life, i.e., pale brown color, setation consisting of long and flattened setae and suberect shorter setae, absence of wings, microphthalmia and elongated legs and antennae. It can be easily separated from the related taxa by the different shapes of the head and profemora and protibiae of the male, and of the aedeagus.

The new synonymy *Tychobythinus cameratensis* (Karaman, 1959) =  *Tychobythinus gracilicornis* (Raffray, 1914) (**syn**. **nov**.) is proposed. New data on morphology, taxonomy and/or distribution of *T. andreinii* (Dodero, 1919), *T. controversus* Poggi 1923, *T. dentimanus* (Reitter, 1884), *T. foroiuliensis* Pace, 1976, *T. glabratus* (Rye, 1870), *T. gladiator gladiator* (Reitter, 1885), *T. majori* (Holdhaus, 1905), *T. mirandus* (Dodero, 1919), *T. myrmido* (Reitter, 1882) and *T. tibialis* (Dodero, 1919) are provided.

## Introduction

In a modern context, the definition of troglobiont species should be broadened to include animals that, while primarily collected in caves, also inhabit the “Milieu Souterrain Superficiel” (MSS). This habitat is interconnected with both the deep hypogean environment – caves and deep rock crevices – and the upper soil horizons [1].

The presence of Pselaphinae in cave has been documented for over 160 years, although these beetles are less frequently collected compared to other cave-dwelling arthropods. This is largely due to their small size (1 to 4 mm), which makes them harder to capture in cave environments [2].

Currently, approximately 170 species of Pselaphinae species are known to be strictly cave-associated [3] and are divided into several diverse tribes, including Euplectini, Trogastrini, Brachyglutini, Iniocyphini, Trichonychini (including old Raffrayiini), Thaumastocephalini, Speleobamini, Tyrini, Pselaphini (one species recently described by Hernando & Castro [4]), Amauropini, Batrisini and Bythinini. The latter two tribes account for over two-thirds of the taxa [2].

The Bythinini tribe is, however, the richest in troglobitic genera and species, with at least 14 genera (many monospecific) exclusively associated with caves. These include *Machaerites* L. Miller 1855, endemic to Italy (Friuli region), Croatia and Slovenia; *Bythoxenus* Motschulsky, 1860, endemic to Italy (Friuli) and Slovenia; *Prionobythus* Jeannel, 1921, from Spain and France; *Leptobythus* Jeannel, 1955, endemic to Majorca island (Spain); *Bathybythus* Besuchet, 1974, from Spain; *Antrobythu*s Besuchet, 1985, from Spain and France; *Gasparobythus* Poggi, 1992, from Italy (Friuli) and Slovenia; *Pauperobythus* Nonveiller, Pavićević & Ozimec, 2002;* Grguria* Pavićević & Ozimec, 2012; *Velebythus* Pavićević & Ozimec, 2012; *Biokovobythus* Pavićević & Ozimec, 2014; *Melledobythus* Hlaváč, Nakládal & Jalžić, 2014, all endemic to Croatia; *Nonveilleria* Pavićević & Besuchet, 2003, from Montenegro and Croatia; and *Speleochus* Park, 1951, from Arkansas and Alabama (United States). It is worth noting that as early as Besuchet ([5]:348); when grouping many Bythinini genera under the single name *Tychobythinus* Ganglbauer, 1896, it was observed that numerous proposed genera emerged as adaptations to endogean or cave life. Only a comprehensive and objective revision of the Bythinini can accurately assess the true taxonomic value of these proposed genera. In our view, the taxonomic status of many of the genera mentioned — often monospecific, endemic, and sympatric — remains unresolved. The validity of these genera remains to be fully established, particularly given that adaptations to endogean or cave life frequently involve pronounced morphological and functional modifications, which can complicate the reconstruction of phylogenetic relationships even among closely related species.

Defining a subterranean or troglobitic taxon also poses challenges. For example, species within the genus *Glyphobythus* Raffray, 1904 exhibit sexually dimorphic traits: females are wingless and either anophthalmic or microphthalmic and generally live in caves, while males are winged and macrophthalmic, allowing them to be found both in caves and in surface (epigean) environments.

Some ecological classifications derive from the rarity of the specimens and the limited knowledge of species behaviors. The case of *Xenobythus serullazi* (Peyerimhoff, 1901), endemic to the Maritime Alpes and Provence (southeastern France) and Piedmont Region (northwestern Italy), is particularly notable. This species, the only representative of its genus, was originally classified as troglobitic until Löbl ([6]:55) reported the capture of a male with well-developed eyes in sifted forest litter.

Later, Orousset & Rougon ([7]:146–147) reported the capture of a winged male in window traps, highlighting that while females of *X. serullazi* are all microphthalmic, with only one ommatidium, and nearly apterous with wings reduced to a tiny hyaline tongue, males show well-developed eyes with 30–36 ommatidia and exhibit either brachypterous or fully macropterous wings, with no intermediate forms.

The two Bythinini genera with the highest species richness are *Bryaxis* Kugelann, 1794 (one of the largest pselaphine genera, with over 385 species and 40 subspecies found across the Palearctic region; see Sabella & Nicolosi [8] and the aforementioned genus *Tychobythinus*. These genera include clearly troglobitic species exhibiting numerous adaptations to subterranean life, such as depigmentation and thinner cuticle, winglessness, microphthalmia or anophthalmia, and elongated appendages (antennae, legs, and palpi).

Some species are found in a variety of non-hypogean habitats, such as woodlands and scrub (where they can be collected from leaf litter, moss, or rotting wood), humid areas (like ponds, marshes, and river floodplains), plant debris piles, nests of micromammals, birds or insects (particularly ants and termites), and occasionally open areas among grass roots or under stones. Species inhabiting these latter environments lack subterranean adaptations: they have a thick and pigmented cuticle, appendages that are generally not particularly elongated, and many retain wings and well-developed eyes.

Focusing on the genus *Tychobythinus* Ganglbauer, 1896, it currently comprises 110 species [9–12], most of which are found in the Palearctic region. Among these, five species are recorded from the northeastern United States, along with one from Taiwan and another from Thailand.

In the Palearctic, *Tychobythinus* species are widely distributed [13], with representatives found in North Africa, southern and Central Europe, the Caucasus, Japan, far eastern Russia, and southeastern China. Most species within the genus are endemic to relatively small areas, a distribution pattern reflecting their specialized ecological niches and limited dispersal abilities, especially in subterranean habitats [14].

In Italy, 44 species and subspecies of *Tychobythinus* have been documented [11,12,15], including five species recorded in Sicily. These species are frequently collected by sifting through deep litter layers and soil, though several are found exclusively in subterranean habitats. Many exhibit varying degrees of microphthalmia and other adaptations to subterranean life [2,16,17].

Sicily is a unique region where carbonate, evaporite, and volcanic subterranean habitats coexist simultaneously. These habitats are of great value due to their diverse geological formations and the unique features of the species they support, which hold high biogeographic significance [18,19]. However, our understanding of subterranean environments in the region remains limited, as research activities have been constrained over the years, partly due to the difficulties in sampling these habitats, which are often hard to access and study [20,21].

Recent extensive research in Sicilian caves has yielded several significant discoveries (e.g., [8]). Among these is *T. muxari* n. sp., a new subterranean species endemic to Ciavuli cave (Sant’Angelo Muxaro, Agrigento), which is presented in detail in this work. Comprehensive illustrations of key diagnostic features are provided, based on observations from both male and female specimens.

Furthermore, we propose the new synonymy: *Bythinopsis cameratensis* Karaman, 1959 =  *Tychobythinus gracilicornis* (Raffray, 1914) (**syn**. **nov**.). Additional data are also presented on the distribution and on morphological variability of certain *Tychobythinus* species from Italy.

## Materials and methods

This study is based on the examination of 163 specimens preserved in the following collections (relevant curator/collection manager are acknowledged in parentheses):

**DBUC** Department of Biological, Geological and Environmental Sciences, University of Catania, Italy (G. Sabella).
**MCZR** Museo Civico di Zoologia di Roma (V. Vomero).
**MHNG** Muséum d’Histoire Naturelle, Genève, Switzerland (G. Cuccodoro).
**MNHN** Muséum National d’Histoire Naturelle, Paris, France (A. Mantilleri).
**MSNV** Museo Civico di Storia Naturale di Verona (L. Latella and R. Salmaso).
**SDEI** Senckenberg Deutsches Entomologisches Institut (V. Ferreira and M. Schroeter).

### Measurements

All the specimens were measured in their dry-mounted state, and the measurements represent the range across all specimens. The body length is measured from the anterior clypeal margin to the posterior margin of the last visible abdominal tergite. The length and width of body parts were measured between points of maximum extension, e.g., the head length is measured between the anterior clypeal margin and the posterior margin of the neck; the head width includes the eyes, the elytral length along the suture line, and the elytral width is the total width of the two elytra taken together. The abdominal tergites are numbered based on their order of visibility. Morphological terminology follows Chandler [22], with the exception that the abdominal sternites are referred to as ventrites here.

The holotype specimen was left intact, while the aedeagus of a male paratype was mounted in Canada balsam. A camera lucida mounted on a Leica DMLB stereomicroscope was used for drawings, while photos were made using a Leica digital camera mounted on Leica DMLB stereomicroscope, using software CombineZ. The scanning electron microscopy (SEM) photos were taken using a Phenom XL G2.

### Nomenclatural acts

The electronic edition of this article conforms to the requirements of the amended International Code of Zoological Nomenclature, and hence the new names contained herein are available under that Code from the electronic edition of this article. This published work and the nomenclatural acts it contains have been registered in ZooBank, the online registration system for the ICZN. The ZooBank LSIDs (Life Science Identifiers) can be resolved and the associated information viewed through any standard web browser by appending the LSID to the prefix “http://zoobank.org/”. The LSID for the nomenclatural act is: urn:lsid:zoobank.org:act:0D5995FF-B29A-4BEB-8888-87276472B040. The LSID for this publication is: urn:lsid:zoobank.org:pub:F85864BA-75EF-42DA-BBF6-102465ECB3A4. The electronic edition of this work was published in a journal with an ISSN, and has been archived and is available from the following digital repositories: PubMed Central, LOCKSS.

### Notes on sampling environment

The karst system of “Sant’Angelo Muxaro” is located in central Sicily, within the municipality of Sant’Angelo Muxaro, Agrigento province (Italy). This area is characterized by evaporitic rocks from the “Gessoso-Solfifero” formation, which dates back to the Messinian age [23,24].

The geological deposits primarily consist of thick beds of selenitic gypsum, along with laminated and detrital gypsum, interspersed with thin layers of gypseous marls, marls, and evaporitic carbonates.

The gypsum layers typically rest on clays, marly clays, and sandy deposits from the middle to lower Serravallian, and in some areas, overlie diatomites and Tripoli marls from the lower Messinian.

The system includes two sinkholes: the ‘Grotta di Sant’Angelo Muxaro’ (also known as ‘Grotta Ciavuli’) and the ‘Inghiottitoio Infantino’. These sinkholes are located at the ends of two blind valleys at the base of the southern slope of the hill on which the town of Sant’Angelo Muxaro stands. An active resurgence is also present in a cave on the northwestern slope of the same hill [25].

**Fig 1:**
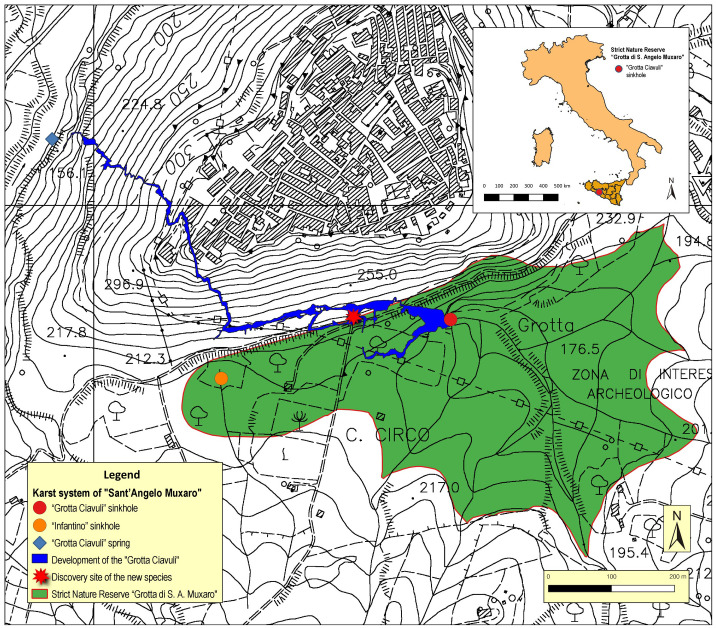
Location map of “Riserva Naturale Integrale Grotta di Sant'Angelo Muxaro”.

Since 2000, the “Grotta Ciavuli”, the “Inghiottitoio Infantino”, and a segment of their feeding basin, totaling approximately 21 hectares, have been designated as a nature reserve. This reserve is officially recognized as the “Riserva Naturale Integrale Grotta di S. Angelo Muxaro” and is registered under EUAP1098 in the Official Register of Protected Natural Areas. Administered by Legambiente Sicilia, the reserve has safeguarded these locations. Moreover, since 2015, these three cavities have received additional protection under the designation of the comprehensive geosite known as the “Sistema carsico di Sant’Angelo Muxaro” (Fig 1).

**Fig 2:**
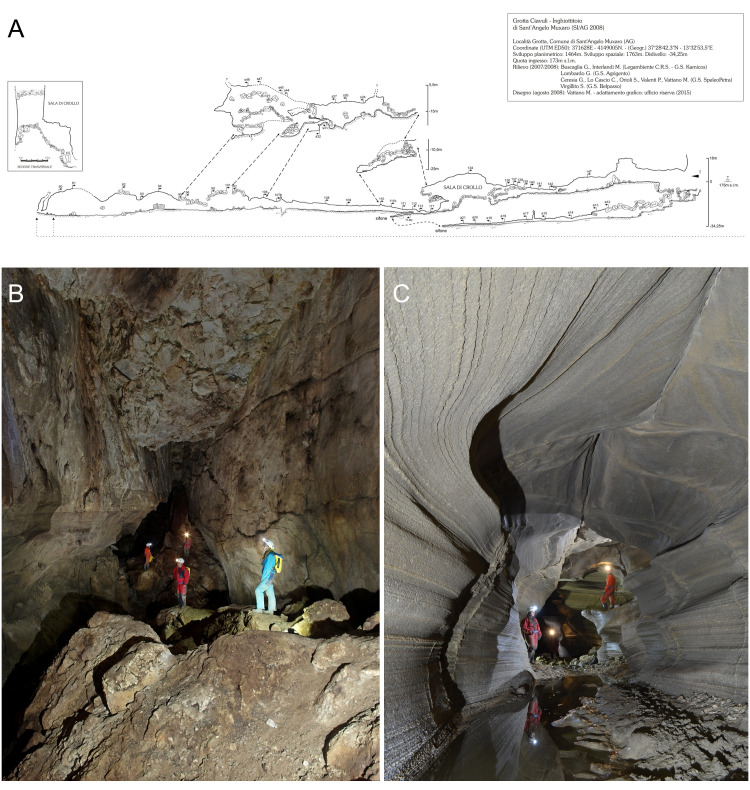
Topography of the “Grotta di Sant’Angelo Muxaro” or “Grotta Ciavuli” (adapted from Vattano, 2008) (A). The “Sala Grande” within the inactive second branch of “Grotta di Sant’Angelo Muxaro”. Photo: G. Buscaglia (B). The active first branch characterized by external water inflows. Photo: M. M. Interlandi (C).

The primary cavity in the system is the “Grotta di Sant’Angelo Muxaro” (cadastral number 2008SI-AG), characterized by a planimetric development of 1460 m, a spatial development of 1760 m, and a vertical drop of -34 m [25]. Water enters the cave at an altitude of approximately 175 meters, forming a small siphoning lake in the terminal part of the cavity. The cave, surveyed last in 2008 [26], follows discontinuity lines predominantly in the E-W and N-S directions, with subordinated NE-SW orientations, dividing into two main branches. The active first branch channels external water contributions, leading to a small chamber with a lake. The gallery extends to a siphoning lake, connecting to the second portion. The inactive second branch, except for rare meteoric events, is the well-known part of the protected site. Accessible through a wide entrance cavity, it leads to inner environments where specimens for this study were found via two overlapping passages. Here, several specimens of the new species were collected while examining deposits of organic material originating from outside the cave, such as twigs, leaves, and other organic materials (Fig 2).

## Results

### Description of a new species

**Fig 3:**
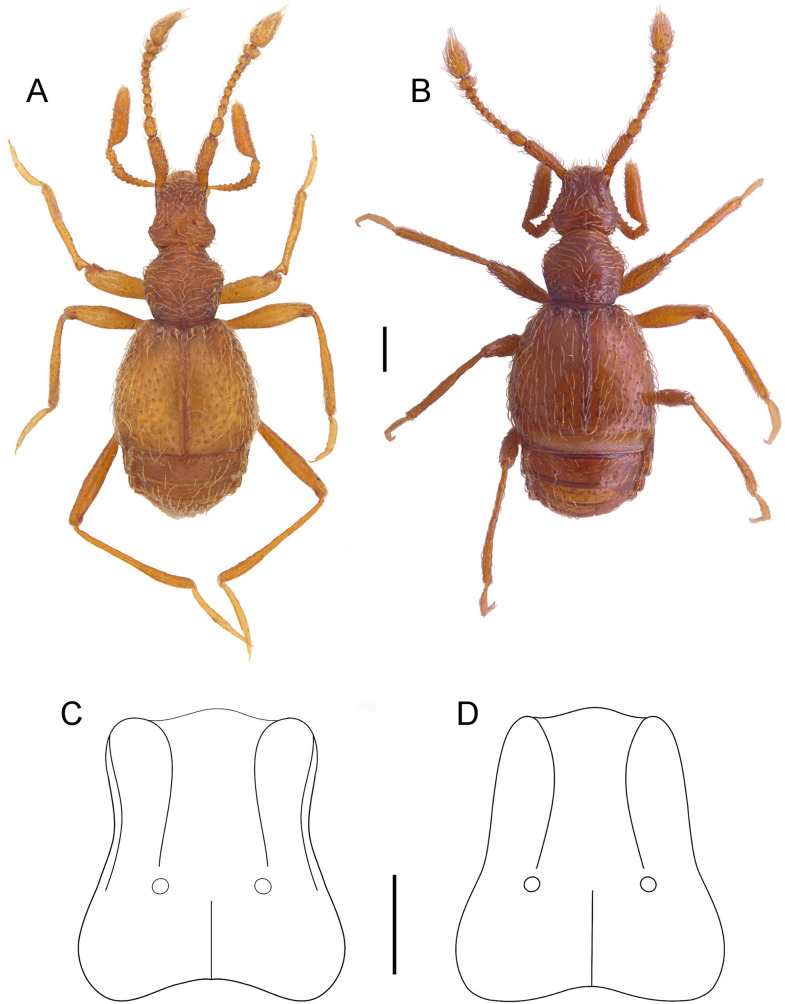
*Tychobythinus* *muxari* n. sp., dorsal view. Holotype male, habitus (A). Paratype female, habitus (B). Head of male, schematic drawing (C). Head of female, schematic drawing (D). Scale bar 0.1 mm.

**Fig 4:**
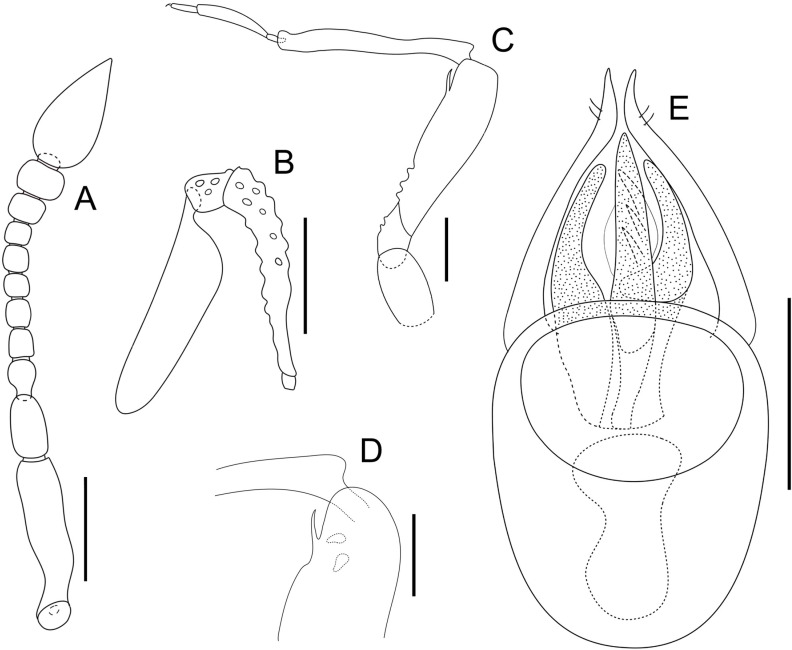
*Tychobythinus* *muxari* n. sp., paratype male. Left antenna, dorsal view (A). Right maxillary palpus, dorsal view (B). Right anterior leg, lateral view (C). Particular of profemur and protibia, lateral view (D). Aedeagus, dorsal view (E). Scale bar 0.1 mm.

**Fig 5:**
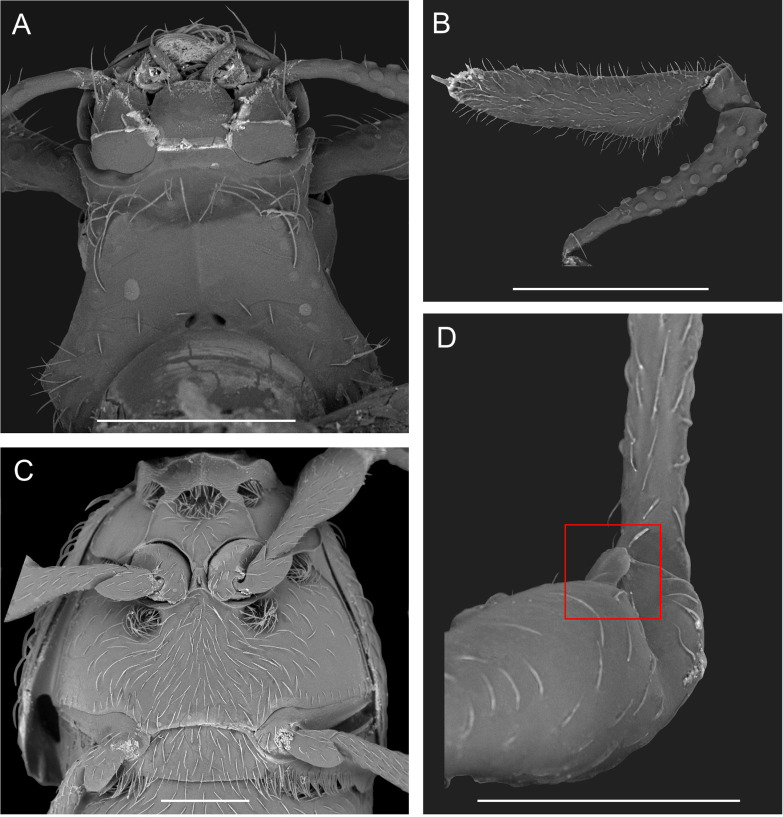
*Tychobythinus* *muxari* n. sp., paratype male, SEM imagines. Head, ventral view, scale bar 150 μm (A). Right maxillary palpus, dorsal view, scale bar 100 μm (B). Mesoventrite and metaventrite, ventral view, scale bar 150 μm (C). Particular of profemur and protibia, lateral view, scale bar 80 μm (D).

***Tychobythinus muxari* Sabella, Interlandi and Nicolosi n. sp.** urn:lsid:zoobank.org:act:0D5995FF-B29A-4BEB-8888-87276472B040 (Figs 3–5)

**Type material**. **Holotype**: ITALY: **Sicily Region**: *Agrigento* province: Sant’Angelo Muxaro (AG), Ciavuli Cave, 18.X.2023, 1 ♂, G. Nicolosi leg. (DBUC).

**Paratypes (5 ex.)**: ITALY: **Sicily Region**: *Agrigento* province: 2 ♂♂ and 1 ♀, same data as holotype (DBUC); 1 ♀, same locality, 29.I.2023, G. Nicolosi leg. (DBUC); 1 ♀, same locality, 14.IX.2023, G. Nicolosi leg. (DBUC).

**Description***. Male* (Fig 3A): Length 1.35–1.40 mm, apterous and microphthalmous. Entirely yellowish including antennae, palpi, and legs. Dense pubescence consists of long and flattened setae (length: 0.05–0.06 mm) on head, pronotum, elytra and abdomen, very dense at the base of first abdominal visible sternite; suberect shorter setae (length: 0.02–0.03 mm) present on the base of the antennae, on legs, and last palpomere.

Head (Fig 3C) slightly wider (0.26 mm) than long (0.25–0.255 mm) distinctly narrower than pronotum. Eyes reduced, with only 2–3 ommatidia. Frontal lobe 0.195–0.20 mm wide with subparallel, protruding and sharp side; antennal tubercle protruding. Convex occipital region traversed by a median longitudinal carina reaching about to posterior edge of vertexal foveae. The latter relatively wide and well-impressed. Surface of frons flattened and punctuated, the frons between antennal tubercles with large median sulcus reaching the anterior edge of vertexal foveae. Clypeal carina well-defined, equally visible in dorsal as well in lateral view, extends to the ocular region. Tempora slightly rounded. Gular region (Fig 5A) with a pair of gular foveae; its surface with some flat tubercles and hollowed behind labium. This depression margined posteriorly by a not very prominent transverse ridge bearing some setae.

Antennae (Fig 4A) 0.79–0.80 mm long, scape about  three times as long (0.175–0.18 mm) than wide (0.055–0.061 mm), narrowed and flattened with protruding and sharp medial margin in basal third, wider in middle, its surface with some tubercles. Pedicel slightly asymmetric, about one and a half times longer than wide, slightly tighter than scape, and wider than funicular segments. Antennomere III longer than wide and slightly narrowed at base; antennomere IV as long as wide, antennomere V longer than wide, antennomeres VI–VII subequal and as long as wide, antennomere VIII wider than long. Antennal club consisting of last three antennomeres which are broaden progressively from IX to XI. Antennomere IX slightly wider than long, antennomere X distinctly wider than long, antennomere XI distinctly longer than wide and twice as long as combined length of antennomeres IX and X. Maxillary palpi (Figs 4B and 5B) with palpomere II elongated and gradually expanded from base to apex, its surface covered by 20–24 tubercles. Palpomere III slightly longer than wide, its surface with 6–8 tubercles; last palpomere about 3.5 times longer (0.22–0.23 mm) as wide (0.06 mm), widest in basal third, its lateral margin right.

Pronotum slightly wider (0.31 mm) than long (0.30 mm), with slightly convex disc, its surface with only some punctures; the maximum wide of pronotum about at the middle, anterior portion of lateral margins slightly convergent and sinuates, posterior portion of lateral margins almost straight. Evident pleural carina oblique, well-defined. Two well impressed antebasal lateral fovea linked by the antebasal sulcus. Tegument between pronotal posterior margin and antebasal sulcus rough, it makes difficult to recognize median antebasal fovea. Mesoventrite and metaventrite as in Fig 5C, with median egg-shaped impression beginning from its posterior margin and extending just before mesocoxal cavities, and bearing for its entire length a median longitudinal carina. Base of mesocoxal cavities with pubescent pit on each side.

Elytra distinctly wider (0.56–0.57 mm) than long (0.53 mm), convex, sides slightly rounded from base to the apex, widest near the middle. Humeral callus strongly reduced. Dorsal surface with some punctures. Each elytron with two big basal foveae, subhumeral fovea well-defined. Both marginal and sutural striae reaching elytral apex, discal striae lacking.

Abdomen normally shaped without particular characters.

Legs relatively long and thin. Protrochanters and profemora with some tubercles on ventral surface, profemora swollen with ventral face of the distal third excavated, the latter is surmounted by a large median apophysis rounded at the apex (Figs 4C–D and 5D); protibiae slightly flattened and enlarged in the distal third, the lateral margin of protibiae strongly enlarged and rounded in basal portion. Meso and metatrochanters simple; meso and metafemora slightly swollen, mesotibiae simple, length of metatibiae: 0.51–0.52 mm, slightly enlarged and sinuate in the distal third.

Aedeagus (Fig 4E) 0.28–0.285 mm long, ovoid with relatively long parameres, that are convergent and with narrow apex, each bearing two subapical setae. Internal sac with 2 lateral big and stout teeth and a median tooth with 3 internal spines.

*Female* (Fig 3B): Similar to male but head (Fig 3D) wider (0.26–0.265 mm) than long (0.225–0.23 mm) with frontal lobe narrower than in male (0.17–0.175 mm wide) with lateral sides slightly convergent, its surface with only some punctures, shorter antenna (0.67–0.68 mm), gular region unmodified, slightly convex. Metaventrite lacking median impression and longitudinal carina; unmodified legs.

## Discussion

**Fig 6:**
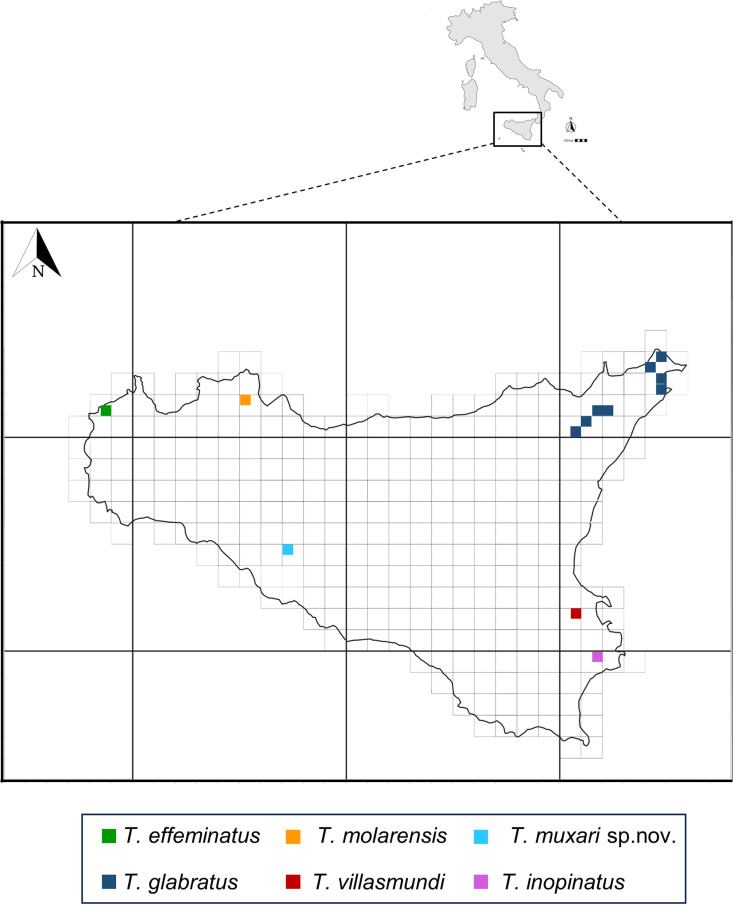
Distribution in Sicily of the species of the genus *Tychobythinus.*

Through extensive fieldwork in these cave environments in Sicily region, a new species of *Tychobythinus* has been discovered, described in this study. The genus now comprises six species in Sicily: 1) *T. glabratus* (Rye 1870), widely distributed in Western Europe, and reported in Sicily only from the Peloritani Mountains [27]; 2) *T. effeminatus* Sabella 1999, strictly endogean and endemic to Sicily, known only from Mount San Giuliano (Erice, Trapani); 3) *T. molarensis* Sabella, Grasso & Spena 2012, a troglobitic species from Molara Cave (Palermo, Palermo province); 4) *T. villasmundi* Sabella, Amore & Nicolosi 2019, a troglobitic species endemic to Villasmundo Cave (Melilli, Syracuse province); 5) *T. inopinatus* Sabella, Costanzo & Nicolosi, 2020, a troglobitic species endemic to Monello Cave (Syracuse, Syracuse province), and 6) *T. muxari* n. sp., a troglobitic species endemic to Ciauli Cave (Sant’Angelo Muxaro, Agrigento province); for the distribution in Sicily of these species (see also Fig 6).

*T. muxari* n. sp. differs from all other *Tychobythinus* species in its aedeagal and exoskeleton features. It shares some affinities with *Tychobythinus molarensis*, but differs in several characters: the shape of the head both in male than in female (cfr. Figs 1C–D with figures 3 and 5 in Sabella [2], the shorter antennal scape (0.175–0.18 mm in *T. muxari* against 0.19–0.20 mm in *T. molarensis*), the longer last palpomere (0.22–0.23 mm in *T. muxari* against 0.21 mm in *T. molarensis*), the different shape of profemora and protibiae of male (cfr. Figs 4C–D and 5D with figure 8 in Sabella [2]) and finally for the morphology of the internal sac of aedeagus (cfr. Fig 4E with figure 10 in Sabella [2]).

**Fig 7:**
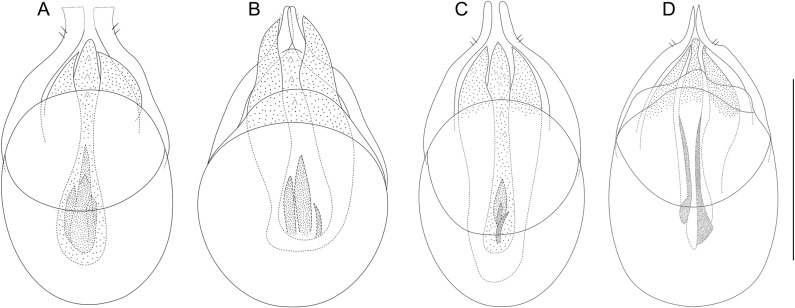
Aedeagi of *Tychobythinus*, dorsal view. *T. andreinii* from Sansa (Salerno province) (A). *T. gracilicornis* from **N** Sella Leonessa (Terminillo Mount, Rieti province) (B). *T. cameratensis* from Camerata Nuova (Roma province) (C). *T. majori* from Monte Penna (Genova province) (D). Scale bar 0.1 mm.

**Fig 8:**
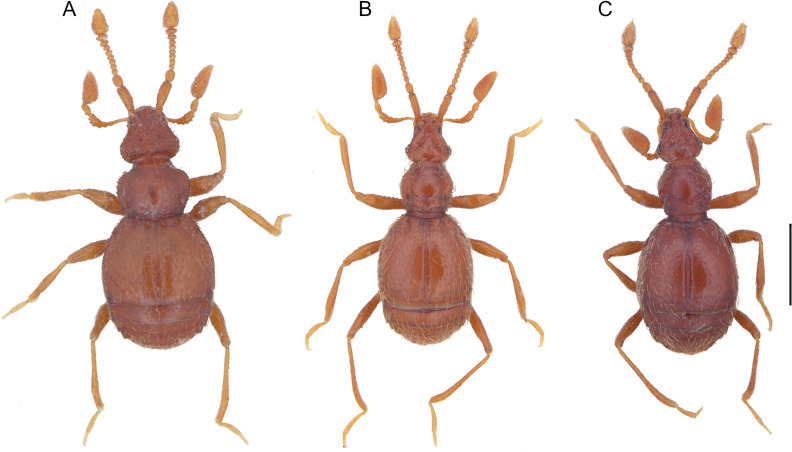
Habitus of *Tychobythinus*, dorsal view. *T. foroiuliensis*, female (A). *T. gracilicornis*, male from **N** Sella Leonessa (Terminillo Mount, Rieti province) (B). *T. cameratensis*, male from Camerata Nuova (Roma province) (C). Scale bar: 0.1 mm.

**Fig 9:**
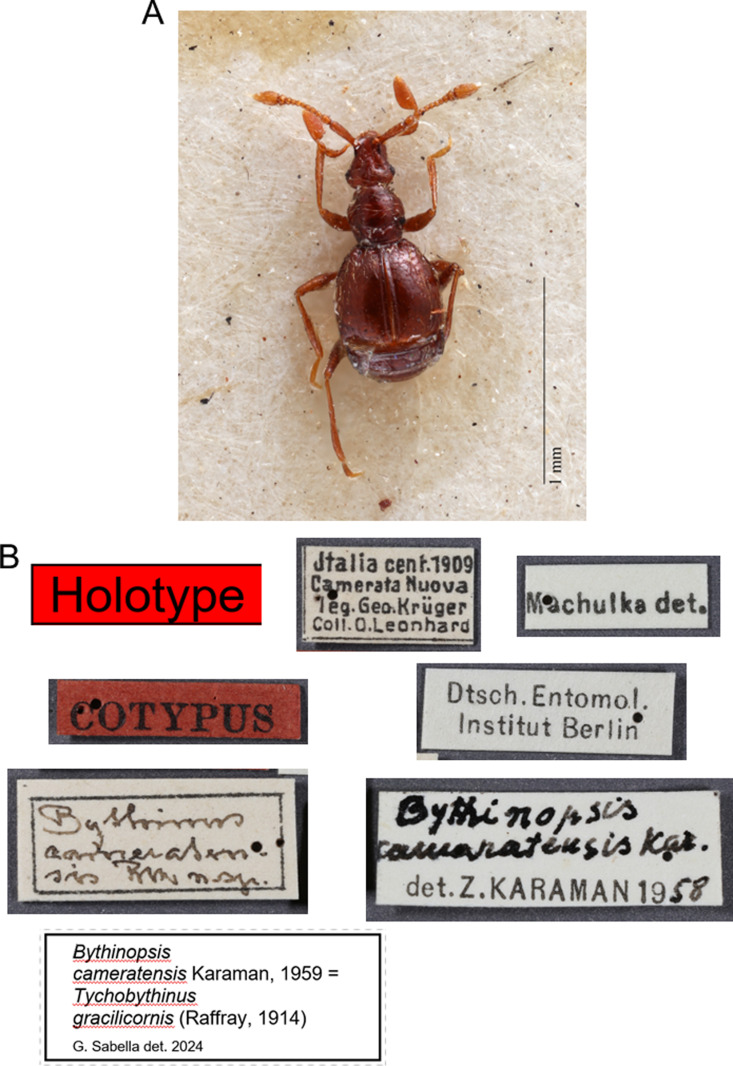
*Tychobythinus cameratensis,* holotype. Habitus, dorsal view (A). Labels of holotype (B).

**Fig 10:**
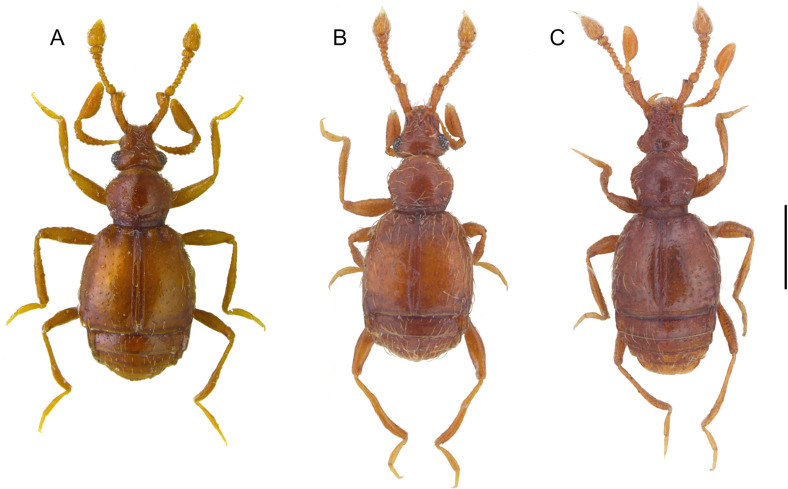
Habitus of male of *Tychobythinus majori*, dorsal view. Specimen from Elba Island (Livorno province) macrophthalmos with modified antennal scape and unmodified metatibiae (A). Specimen from Elba Island (Livorno province) macrophthalmos with unmodified antennal scape and modified metatibiae (B). Specimen from Monte Penna (Genova province) microphthalmos with modified antennal scape and unmodified metatibiae (C). Scale bar 0.1 mm.

This new discovery in Sicily’s subterranean habitats emphasizes the need to intensify sampling efforts in the region. Such efforts are crucial not only for the conservation of these largely unexplored environments but also for safeguarding their highly valuable habitats, which face significant threats from direct and indirect anthropogenic disturbances, including intensified land use, wildfires, quarrying, urbanization, and the construction of rural homes and infrastructure [28].

### New data on some Italian species of *Tychobythinus*

Given our recent examination of numerous Italian specimens of different species within the genus *Tychobythinus*, we are now taking the opportunity to update the taxonomic and/or geonymic data and propose a new synonymy.

#### *Tychobythinus andreinii* (Dodero, 1919) (Fig 7A).

**Material examined (7 ex.)**: ITALY – **Campania**: *Salerno* province: Sansa (Cilento), Bosco Centaurino, 27.VI.1994, oak forest, sifting litter, F. Angelini, 1 ♂ (DBUC); **Basilicata**: *Potenza* province: Lagonegro, Ponte della Calda, 21.VI.1991, oak forest, F. Angelini, 1 ♂ (DBUC); **Calabria**: *Cosenza* province: Orsomarso, Argentina valley, 15.VI.1991, oak forest, F. Angelini, 3 ♂♂ (DBUC); *Crotone* province: Verzino, 750 m, 13.IV.2018, oak forest sifting litter, G. Sabella, 1 ♀ (DBUC); *Reggio Calabria* province: Piani of Aspromonte, 1000 m, 14.VI.1991, F. Angelini, 1 ♀ (DBUC).

Species so far known for Basilicata and Calabria [15] now also reported for Campania and the aedeagus of the male from Sansa (Salerno province) is showed in Fig 7A.

#### *Tychobythinus controversus* Poggi, 2023.

**Material examined (1 ex.)**: ITALY – **Sardinia**: *Sassari* province: environs of Sassari, 08.IV.1977, at the base of the olive tree, S. Vit, 1 ♂ (MHNG).

Species recently described by Poggi ([11]: 448, Figs 11 and 12) and known only for the male holotype collected by Dodero on 14.III.1912 in Macomér (Nuoro province). The specimen in question is identical to the holotype for the morphology of the profemur and the protibia while the aedeagus presents some small differences in the armor of the internal sac which however can be made to fall within normal intraspecific variability.

#### *Tychobythinus dentimanus* (Reitter, 1884).

**Material examined (2 ex.)**: ITALY – **Sardinia**: *Cagliari* province: Monte Rocca Steria (Sulcis), 900 m, III.1983, under stones, granite, 1 ♂ (MHNG); *Sud Sardegna* province: Carbonia Iglesias, Gonnesa Fontanamare, 27.I.2011, *Pistacia lentiscus,* C. Bellò, 1 ♂ (MHNG).

Sardinian-Corsican endemic species, distributed and relatively frequent throughout Sardinia ([11]: 440). The males are characterized by a significant variability in the morphology of the profemora and protibiae and also in the armor of the internal sac of the aedeagus, but this variability does not affect their recognition and their specific attribution.

#### *Tychobythinus foroiuliensis* Pace, 1976 (Fig 8A).

**Material examined (1 ex.)**: ITALY – **Friuli-Venezia-Giulia**: *Pordenone* province: Montereale Valcellina, 10.III.1990, P. Visentini, 1 ♀ (DBUC).

A strictly Friulian endemic species known so far only for the province of Pordenone where it was collected not only in the typical locality (northern slope of Monte Jouf, Maniago) but also on the northern slope of Monte Ciaurlec (Battei, Pradis di Sotto). The habitus of female is shown in Fig 8A.

#### *Tychobythinus glabratus* (Rye, 1870).

**Material examined (96 ex.)**: ITALY – **Lombardy**: *Pavia* province: Oriolo (Voghera), IX.1985, A. Solari, 4 ♂♂ and 1 ♀ (MHNG); **Veneto**: *Treviso* province: Ponzano Veneto, IV.1959, M. Burlini, 1 ♂ and 1 ♀ (MHNG); same data but XI.1934, 1 ♂ and 1 ♀ (MHNG); *Venezia* province: Caposile, 23.III.1958, B. Cadamuro, 1 ♂ (MHNG); San Giuliano, 28.II.1954, I. Bucciarelli, 1 ♂ (MHNG); *Vicenza* province: Belvedere (Tezze sul Brenta), 17.VII.1910, J. Pinker, 1 ♂ (MHNG); *Verona* province: Musella Park, Loara valley, *Quercus robur*, 09.IV.1991, A. Zanetti, 1 ♀ (MSNV); Eremo San Felice (Cologna Veneta), 20.IV.1896, A. Fiori, 1 ♂ and 1 ♀ (MHNG); **Liguria**: *La Spezia* province: environs of Bonassola, *Quercus ilex*, 07.IX.1983, S. Zoia, 1 ♂ and 2 ♀♀ (MSNV); *Genova* province: Gravelia valley, Piandifieno (Ne), 03.VIII.1980, S. Zoia, 1 ♀ (MHNG); environs of Prato, 10.III.1976, *Quercus ilex*, S. Zoia,1 ♀ (MHNG); Gravelia valley, 08.VIII.1976, L. Briganti, 1 ♀ (MHNG); Lavagna, 27.VI.1976, S. Zoia, 3 ♀♀ (MHNG); Rio S. Giulia valley (Lavagna), 22.X.1976, S. Zoia, 1 ♂ (MHNG); same locality, S. Zoia, 26.XII.1976, 2♀♀ (MHNG); **Marche**: *Ancona* province: Genga, VI.1937; M. Lombardi, 2 ♀♀ (MSNV); Monte Conero, G. Paganetti, 1 ♂ and 2 ♀♀ (MHNG); *Pesaro and Urbino* province: Monte Nerone (Cagli), 24.VI.1976; M. Curti, 1 ♂ (MHNG); **Tuscany**: *Florence* province: environs of Florence, IX.1923, M. Lombardi, 1 ♀ (MSNV); *Livorno* province: Collesalvetti, G. Paganetti, 1 ♀ (MHNG); Elba Island, north slope of Monte Capanne, 5 ♂♂ and 2 ♀♀ (MHNG); **Latium**: *Latina* province: Palmarola Island, Fosso di Radiche, 14.XI.1966, R. Argano, 1 ♂ (MCZR); Zannone Island, Macchia alta, 28.II.1966, R. Argano, 1 ♂ (MCZR); Sanctuary of the Madonna of Civita (Itri), 665 m, 03.V.1996; F. Angelini, 2 ♂♂ and 3 ♀♀ (DBUC); *Roma* province: Marino, M. Cerruti, IV.1928, 1 ♀ (MSNR, ex coll. Cerruti); Frascati, Cerruti, IV.1927, 1 ♂ (MCZR, ex coll. Cerruti); **Campania**: *Benevento* province: Montefusco, 20.XII.1975, M. Curti, 1 ♂ (MHNG); *Salerno* province: slope of Monte Sacro (Cilento), 1000 m, chestnut, 29.VI.1994, F. Angelini, 2 ♂♂ and 1 ♀ (DBUC); Monte Sacro (Cilento), 1650 m, beech forest, 30.VI.1994, F. Angelini, 1 ♂ (DBUC); Sambiase (Ceraso); 14.IX.1901, A. Solari, 1 ♂ (MHNG); **Puglia**: *Bari* province: Monte Sannace (Murge), oak forest, sifting litter, 01.XII.1991, F. Angelini, 1 ♂ (DBUC); Gioia del Colle, 11.X.1981, L. De Marzo, 1 ♀ (MHNG); *Brindisi* province: Monte Canne (Fasano), 17.VII.1980, chestnut, S. Vit, 1 ♂ (MHNG); *Taranto* province: Martina Franca, entrance of the cave Pu846, 21.VIII.1980, S. Vit, 1 ♀ (MHNG); Monte delle Pianelle, 25.VII.1980, litter along stream, S. Vit, 2 ♂♂ (MHNG); same data but rotten wood, 2 ♀♀ (MHNG); Palagiano, 19.VII.1980, foot olive, S. Vit, 1 ♂ and 1 ♀ (MHNG); *Brindisi* province: Torre Canne, 17.VII.1980, carob foot, S. Vit, 1 ♂ (MHNG); Ceglie-Messapica, 16.VII.1980, litter, S. Vit, 1 ♂ and 1 ♀ (MHNG); Casamassima, 03.IX.1982, G. De Marzo 1 ♀ (MHNG); **Basilicata**: *Potenza* province: Lagonegro, Ponte della Calda, 19.XII.1993, oak forest, F. Angelini, 1 ♂ (DBUC); *Matera* province: Sinkhole above Cave of Pipistrelli (Matera), G. Meggiolaro, 24.X.1966, 1 ♂ (MSNV); Policoro, 27.VII.1980, *Rubus*, S. Vit, 2 ♂♂ (MHNG); same data but *Agave*, 1 ♂ (MHNG); same data but litter, 1 ♀ (MHNG); Policoro, 19.VII.1984, S. Vit, 1 ♂ and 1 ♀ (MHNG); same locality, 20.VII.1984, S. Vit, 1 ♀ (MHNG); **Calabria**: *Cosenza* province: Pantanelle (Grisolia, Orsomarso), 700 m, 15.VI.1996, hazelnut grove, F. Angelini, 4 ♂♂ and 6 ♀♀ (DBUC); *Reggio Calabria* province: Antonimina, X.1968, 3 ♂♂ and 1 ♀ (MSNV); Gerace, G. Paganetti, 1 ♂ (MHNG); Stilo, about 400 m, 08.VI.1976; M. Curti, 1 ♂ and 1 ♀ (MHNG).

Species reported for: England, Germany, Belgium, France, Switzerland, Italy, Slovenia and Croatia [29].

In Italy it is known for: Piedmont, Lombardy, Trentino Alto Adige, Veneto, Friuli Venezia Giulia, Liguria, Emilia Romagna, Tuscany, Marche, Umbria, Lazio, Abruzzo, Molise, Campania, Puglia, Basilicata, Calabria and Sicily [15].

#### *Tychobythinus gladiator gladiator* (Reitter, 1885).

**Material examined (17 ex.)**: ITALY – **Emilia Romagna**: *Piacenza* province: Santa Maria, 15.X.1896, A. Fiori, 1 ♂ (MHNG); *Reggio Emilia* province: Santa Maria Maddalena Cave (Valestra, Carpineti), 12.IV.1976, I. Loebl, 1 ♂ and 2 ♀♀ (MHNG); same locality, 02.IX.1978, B. Hauser, 1 ♂ (MHNG); *Ravenna* province: Brisighella, 250 m, 09.X.1984, F. Poggi, 1 ♂ and 1 ♀ (DBUC); **Marche**: *Pesaro and Urbino* province: Bellisio Solfare (Pergola), 25.VI.1974, R. Pace, 1 ♂ (MSNV); Bellisio Solfare (Pergola), 350 m, 25.VII.1974, R. Pace, 2 ♀♀ (MSNV); *Ancona* province.: Genga, IX.1920, A. Dodero, 1 ♂ (MSNV); Genga, A. Martelli, 1 ♂ (MHNG); Sirolo, 01-06.IX.1964, 1 ♀ (MHNG); *Macerata* province: Monte Bicco, 1700 m, 21.VI.1976, M. Curti, 1 ♂ (MHNG); **Tuscany**: *Lucca* province: Cardoso, 300 m, 03.VI.1984, V. and C. Brachat, 1 ♂ (MHNG); Fornovolasco (Fabbriche di Vergemoli), 26.III.1973, L. Briganti, 1 ♂ (MHNG); **Latium**: *Roma* province: Carpineto Romano, Piana Faggio, 1200 m, 06.V.1996, *Fagus*, F. Angelini, 1 ♀ (DBUC).

*Tychobythinus gladiator* (Reitter, 1885) is a species endemic of the southcentral Italian Apennines of which two subspecies are known: *T. g. gladiator* (Reitter, 1885) from Emilia Romagna, Tuscany, Marche, Umbria, Lazio, Campania (Poggi, 2021), and *T. g. miles* (Raffray, 1914) distributed in Lazio and Abruzzo [15].

#### *Tychobythinus gracilicornis* (Raffray, 1914) and *T. cameratensis* (Karaman, 1959).

#### *Tychobythinus gracilicornis* (Raffray, 1914) (Figs 7B and 8B).

**Type material (1 ex.)**: ITALY – Holotype, here fixed: **Latium**: *Rieti* province: MNHN; 1 ♀; label verbatim: “TYPE (red label)// Mt Terminillo/ Vallonina/ P. Raffray VII.1913// *T. gracilicornis*/ A. Raffray det.// Italie/ centrale”.

**Additional material** (**1 ex**.): ITALY – **Latium**: *Rieti* province: MHNG; 1 ♂; Monte Terminillo, N Sella Leonessa, 42° 29.06’N 13° 0054E, 1710 m, 22.V.2003, *Fagus*, M. Schülke.

This species was until now known only from 3 specimens (2 males and 1 female) collected by Pierre Raffray at the beginning of August 1913 under a large, well-buried boulder within the Terminillo forest at around 1600 m above sea level ([30], 1914: 394). Despite careful and detailed research in the MNHN collections and in particular in the Raffray collection, we were able to find only one female, with a red label “TYPE” and a locality label absolutely consistent with what Raffray stated in the original description (see also the paragraph “Type material”). This female corresponds perfectly to the original description of Raffray [30] (l. c.: 394) and for all intents and purposes it must be considered as the holotype of *T. gracilicornis* (Raffray, 1914).

In the MHNG collections we were able to examine a male of *Tychobythinus* collected in Monte Terminillo in the north of Sella Leonessa, a locality located a few kilometers away as the crow flies from Vallonina, the typical locality of *T. gracilicornis*.

This specimen (Fig 8B) corresponds perfectly to the description and the figure of the male of *T. gracilicornis* provided by Raffray [30] in his original description (l. c.: 394, pl. X, Fig 5) and, in our opinion, there is no reason to doubt that it is attributable to *T. gracilicornis*. Of this specimen we represent the aedeagus (Fig 7B), and, as previously stated, this is the first time that the aedeagus of *T. gracilicornis* has been represented.

#### T. *cameratensis* (Karaman, 1959) (Figs 7C, 8C and 9).

**Type material (1 ex.)**: ITALY – Holotype, here fixed: **Latium**: *Roma* province: SDEI; 1 ♂; label verbatim: “Holotype (red label)// Italia cent.1909/ Camerata Nuova/ leg. Geo. Krüger/ coll. O. Leonhard// COTYPUS (red label)/ *Bythinus*/ *cameratensis*/ mihi n. sp.// Machulka det.// Dtsch. Entomol./ Institut Berlin/ *Bythinopsis*/ *cameratensis* Kar./ det. Z. KARAMAN 1958// *Bythinopsis*/ *cameratensis* Karaman, 1959 = *Tychobythinus*/ *gracilicornis* (Raffray, 1914)/ G. Sabella det. 2024”.

**Additional material (1 ex.)**: ITALY – **Latium**: *Roma* province: MHNG; 1 ♂; label verbatim: “Italia cent. 1909// Camerata Nuova/ leg. Geo. Krüger*/* coll. O. Leonhard// *cameratensis* R.tt n. sp./ *cameratensis* Kar./ Cl. Besuchet/ det. X. 1961// *Tychobythinus/ cameratensis* (Karaman) = / *Tychobythinus/ gracilicornis* (Raffray)/ Sabella det. 2024// *Tychobythinus/ gracilicornis* (Raffray) ♂/ Sabella det. 2024.

The species is so far known only from 3 specimens collected in Latium and Umbria and particulary: the holotype male collected in Camerata Nuova (province of Rome), 1 male from Grotta della Mandorla, 60La/RI (Contigliano, province of Rieti) ([31]: 67) and 1 male from Cascata delle Marmore (province of Terni) ([32]: 94).

In the MHNG collections we were able to examine a male of *Tychobythinus* with the same locality label of holotype (see also the paragraph “Type material”).

This specimen (Fig 8C) corresponds perfectly to the description and the figure of the male of *T. cameratensis* provided by Karaman in his original description ([33]: 3–4, figures 2A head, 2B antenna, – 2C-2D gular region, 2E profemur). Of this specimen we represent also the aedeagus (Fig 7C) which is very similar to the one designed by Karaman ([33]: 4, figure 2F) due to the shape of the parameres which are rounded at the apex and to the morphology of the armor of the internal sac of the aedeagus.

The comparison between the habitus of the topotypical male of *T. gracilicornis* (Fig 8B) and that of the topotypical male of *T. cameratensis* (Fig 8C) and the morphology of their aedeagi (cfr. Figs 7B and 7C) demonstrates how they are largely overlapping, despite some slight differences, i.e., the penultimate article of the maxillary palpus of T*. cameratensis* a little wider than that of *T. gracilicornis*, and the aedeagus shorter and with a more rounded basal capsule of *T. gracilicornis* than that of *T. cameratensis*. Furthermore, also the features of gular region of males of *T. gracilicornis* from Terminillo Mount and of *T. cameratensis* from Camerata Nuova are identical. Thanks to the courtesy and availability of colleagues V. Ferreira and M. Schroeter, we were able to examine also the holotype (here fixed) of *T. cameratensis*, preserved in the collections of Senckenberg Deutsches Entomologisches Institut (SDEI) (Fig 9A–B), which is identical in external morphology and for aedeagus to the previously described MHNG specimen. For all these reasons we propose to consider *Tychobythinus cameratensis* (Karaman, 1959) as a junior synonym of *Tychobythinus gracilicornis* (Raffray, 1914) (**syn. nov.**).

#### *Tychobythinus majori* (Holdhaus, 1905) (Figs 7D and 10A–C).

**Material examined (7 ex.)**: ITALY – **Tuscany**: *Livorno* province: Elba Island: Monte Capanne, North slope, East valley under Poggio, 100-200 m, leaves and roots, 30.III.-13.IV.2021, E. Moczarski & O. Scheerpeltz, 1 ♂ (macrophthalmos) (MHNG); Marciana f. Napoleone, 400 m, 13.V.1998, chestnut, F. Angelini 1 ♂ (macrophthalmos) (DBUC); slope of Monte Poppe, 100 m, 13.V.1998, *Quercus ilex*, F. Angelini 1 ♂ (macrophthalmos, metatibiae arcuate) (DBUC); S. Ilario in Campo, Torre S. Giovanni, 400 m, 26.IV.1985, A. Sette, 2 ♂♂ (1 macrophthalmos and 1 microphthlamos, both with arcuate metatibiae) and 1 ♀ (MSNV); **Liguria**: *Genova* province: Monte Penna, 03.X.1979, M. Curti, 1 ♂ (MHNG).

Holdhaus ([34]: 122) in the original description reports that *T. majori* has both macrophthalmic and microphthalmic specimens and believes that the females of this species have the metatibiae arcuate in the distal two thirds, this character was later reiterated always by Holdhaus ([35]: 122-123, figure 6). Castellini ([36]: 225) rightly highlights how, in reality, the specimens with modified metatibiae are males, while the females of *T. majori* show unmodified metatibiae.

It is therefore a species that shows an evident intraspecific variability with macro and microphthalmic individuals, and a polymorphism of the males (which can have (Fig 10A) the antennal scape widened at the distal end (see also Castellini [36]: figure 4) and unmodified metatibiae (see also Castellini [36]: figure 5) or (Fig 10B) antennal scape unmodified (as in female, see also Castellini [36]: figure 6) and metatibiae arcuate in the distal two thirds (see also Castellini [36]: figure 7) this last males were named by Castellini ([36]: 226) *T*. *majori* m. ♂ *irratus*. According to Castellini [36] (l. c.) the males of these two forms present identical aedeagal character.

We examined a male (Fig 10C) microphthalmos collected in Monte Penna (Liguria) with antennal scape enlarged in distal third and with unmodified metatibiae. Although this male shows some slight differences in the morphology of the antennal scape (which is less enlarged distally), the morphology of its aedeagus (Fig 7D) is largely comparable to that of *T. majori*. We therefore attribute this male to *T. majori*, also in consideration of the considerable variability that characterizes this species.

Until now the species was known only from Tuscany (Elba island and Piombino promontory) and, therefore, it is new for Liguria.

#### *Tychobythinus mirandus* (Dodero, 1919).

**Material examined (1 ex.)**: ITALY – **Sardinia**: *Sud Sardegna* province: Fluminimaggiore, 17.III.1912, A. Dodero, 1 ♀ (MHNG).

The species was so far known from only one female specimen (holotype) collected in Fluminimaggiore on 24.III.1912 (Poggi [12]: 464). To this specimen we now add another female, also collected in Fluminimaggiore on 17.III.1912 which we found in the collections of the MHNG. The specimen, lacking an elytron, can easily be attributed to *T. mirandus* due to the opaque and densely punctuate pronotum, a unique character among the congener Sardinian species.

The male of this species still remains unknown.

#### *Tychobythinus myrmido* (Reitter, 1882).

**Material examined (12 ex.)**: ITALY – **Sardinia**: *Sassari* province: Monte Limbara, 15.IV.1977, S. Vit, 1 ♂ (MHNG); Olbia, Padrogiano, 31.V.1995, sifting *Quercus suber* litter, F. Angelini, 1 ♂ (DBUC); Arzachena, 10.III.1979, M. Curti, 1 ♂ (MHNG); Tempio Pausania, III.1979, M. Curti, 3 ♂♂ and 5 ♀♀ (MHNG); *Nuoro* province: Lula, III.1979, M. Curti, 1 ♂ (MHNG).

Sardinian-Corsican endemic species which appears to be more common in Corsica, but which is nevertheless present in all Sardinian provinces ([11]: 446).

#### *Tychobythinus tibialis* (Dodero, 1919).

**Material examined (9 ex.)**: ITALY – **Sardinia**: *Nuoro* province: Monte Spada, 14.IV.1977; S. Vit, 1 ♂ and 7 ♀♀ (MHNG); Lake of Gusana, 12.IV.1972, S. Vit, 1 ♀ (MHNG).

According to Poggi ([11]: 448) is a Sardinian endemic species known only from Gennargentu massif and Barbagia of Seulo and now also from Barbagia of Ollolai.
